# Effective induction of cytotoxic T cells recognizing an epitope peptide derived from hypoxia-inducible protein 2 (HIG2) in patients with metastatic renal cell carcinoma

**DOI:** 10.1007/s00262-016-1915-5

**Published:** 2016-10-18

**Authors:** Wataru Obara, Takashi Karashima, Kazuyoshi Takeda, Renpei Kato, Yoichiro Kato, Mitsugu Kanehira, Ryo Takata, Keiji Inoue, Toyomasa Katagiri, Taro Shuin, Yusuke Nakamura, Tomoaki Fujioka

**Affiliations:** 1 Department of Urology, Iwate Medical University School of Medicine, 19-1 Uchimaru, Morioka, 020-8505 Japan; 2 Department of Urology, Kochi Medical School, Kochi, Japan; 3 Division of Cell Biology, Biomedical Research Center, Graduated School of Medicine, Juntendo University, Tokyo, Japan; 4 Department of Biofunctional Micribiota, Graduated School of Medicine, Juntendo University, Tokyo, Japan; 5 Division of Genome Medicine, Institute for Genome Research, Tokushima University Graduate School, Tokushima, Japan; 6 Section of Hematology/Oncology, Department of Medicine, The University of Chicago, Chicago, IL USA

**Keywords:** Cancer peptide vaccine, Genome-wide expression profile, Cytotoxic T lymphocyte, Renal cell carcinoma

## Abstract

**Purpose:**

Through genome-wide expression profile analysis, hypoxia-inducible protein 2 (HIG2) has previously been identified as an oncoprotein involved in development/progression of renal cell carcinoma (RCC). We subsequently identified a highly immunogenic HLA-A*0201/0206-restricted epitope peptide (HIG2-9-4) corresponding to a part of HIG2 and applied it as a therapeutic vaccine. We conducted a phase I clinical trial using the HIG2-9-4 peptide for patients with advanced RCC.

**Materials and Methods:**

Nine patients having HLA-A*0201 or HLA-A*0206 with metastatic or unresectable RCC after failure of the cytokine and/or tyrosine kinase inhibitor therapies were enrolled in this study. The patients received subcutaneous administration of the peptide as an emulsion form with Montanide ISA-51 VG once a week in a dose-escalation manner (doses of 0.5, 1.0, or 3.0 mg/body, 3 patients for each dose). The primary endpoint was safety, and the secondary endpoints were immunological and clinical responses.

**Results:**

Vaccinations with HIG2-9-4 peptide could be well tolerated without any serious systemic adverse events. Peptide-specific cytotoxic T lymphocyte (CTL) responses were detected in eight of the nine patients. Doses of 1.0 or 3.0 mg/body seemed to induce a CTL response better than did a dose of 0.5 mg/body, although the number of patients was too small to draw a firm conclusion. The disease control rate (stable disease for ≥4 months) was 77.8 %, and the median progression-free survival time was 10.3 months.

**Conclusions:**

HIG2-9-4 peptide vaccine treatment was tolerable and effectively induced peptide-specific CTLs in RCC patients. This novel peptide vaccine therapy for RCC is promising.

## Introduction

The majority (70 %) of patients with renal cell carcinoma (RCC) and metastatic disease are managed non-surgically, whereas approximately 30 % undergo cytoreductive nephrectomy [1, 2]. The classical standard therapy for metastatic RCC (mRCC) was cytokine-based immunotherapy with IL-2 and/or IFN-α that exhibited few durable complete remissions [3, 4]. Molecular-targeted therapies have been recently developed as novel standard treatments for mRCC. However, despite their significant anti-tumor effects, only a small subset of patients can be cured and the majority of patients suffer from very severe adverse reactions, including hand-foot syndrome, liver dysfunction, and interstitial pneumonia. Therefore, development of a novel anti-cancer drug for mRCC is eagerly expected.

Since RCC is one of the most immunoresponsive cancers in humans, immunotherapy has been considered to be a promising treatment strategy against RCC. Progress in biomedical research over the last two decades has provided several options for cancer immunotherapy. These include non-specific immune activation by cytokines, passive immunotherapy with specific antibodies to block immune checkpoint molecules, active immunotherapy that induces cytotoxic T cells with oncoantigen peptides or neoantigens [5], and T cell receptor-engineered adaptive immunotherapy [6]. Cancer peptide vaccines are comprised of short or long amino acid sequences as tumor antigens combined with a vaccine adjuvant. Thus, they fall broadly into the category of defined antigen vaccines. In this study, we applied a novel therapeutic peptide vaccine derived from hypoxia-inducible protein 2 (HIG2) to activate RCC-specific cytotoxic T lymphocytes (CTLs).

We previously reported HIG2 as an oncofetal protein that was highly expressed in RCC and fetal kidney as determined by genome-wide expression profile analysis [7]. Because HIG2 expression was specific to RCC and had an expression that was hardly detectable in normal organs, we considered HIG2 to be a good candidate for the development of molecular-targeted therapies against RCC. As one of the approaches to develop anti-HIG2 drugs, we screened and identified a human leukocyte antigen (HLA)-A*0201/0206-restricted epitope peptide, named HIG2-9-4 peptide that could have a high antigenic activity to induce CTLs [8]. So, far, several peptide epitopes derived from cancer-testis antigens or oncofetal proteins have been investigated in translational research targeting several types of human cancer [9–17]. We conducted a Phase I clinical trial using the HIG2-9-4 peptide vaccine for patients with metastatic RCC.

## Materials and methods

### Peptides

HIG2-derived 9- and 10-mer peptides that have high binding affinity to HLA-A:*02:01 were identified as candidates by using the binding prediction software, BioInformatics and molecular analysis section (BIMAS), and the homologous sequences were screened by using the homology search program, basic local alignment search tool (BLAST), as previously reported [8]. The selected high-affinity peptide, HIG2-9-4 peptide (VLNLYLLGV), was manufactured as good manufacturing practice (GMP) grade at a purity of >95 % for the clinical trial by the American Peptide Company Inc. (Sunnyvale, CA). HLA-A*0201-restricted HIV-derived epitope peptide (ILKEPVHGV) was also synthesized by American Peptide Company Inc., for control measurements of the CTL response.

### Clinical study design

This study was a non-randomized, open-label, phase I clinical trial involving dose-escalation of the HIG2-9-4 peptide for patients with advanced RCC. The primary endpoint was safety of this novel peptide vaccine, and the secondary endpoints were immunological responses, clinical outcomes, and the determination of the optimal dose for further clinical trials. Immunological responses were evaluated by measuring interferon (IFN)-γ production from specific CTLs responding to the HIG2-9-4 peptide and immunological reactions at the injection sites (RAI). RAI was defined by erythema and/or induration at the injection site of the peptide vaccine. Clinical outcomes were assessed by computed tomography (CT) scanning results in accordance with the Response Evaluation Criteria in Solid Tumors (RECIST) criteria (version 1.1), progression-free survival (PFS), and overall survival (OS). CT scanning was performed within 1 month before the vaccination and after every course of the vaccination. PFS and OS curves were estimated by using Kaplan–Meier methodology. PFS was determined as the time from the date of the initial vaccination until the decision of disease progression. OS was calculated from the date of the initial vaccination to the date of death. This trial was registered in ClinicalTrials.gov (No. NCT01254838).

### Patient eligibility

Patients with pathologically confirmed clear-cell RCC that progressed after standard therapy, such as surgical resection, cytokine treatment, or molecular-targeted therapy (sorafenib or sunitinib), were enrolled in this trial from November 2008 to August 2009 at Iwate Medical University and Kochi Medical School. The eligibility criteria were (1) unresectable mRCC, recurrent and/or locally advanced disease diagnosed by imaging analysis, (2) HLA-A*0201 or 0206 genotype, (3) an Eastern Cooperative Oncology Group (ECOG) performance status of 0–2, (4) age 20–79 years and life expectancy of ≥3 months, (5) adequate hepatic, renal, and bone marrow function (white blood cell count of ≥2000/μL, platelets of ≥75,000/μL, aspartate aminotransferase of ≤50 IU/L, alanine aminotransferase of ≤150 IU/L, total bilirubin of ≤3 g/dL, and serum creatinine of ≤1.5 mg/dL), and (6) no other immunotherapy, molecular target therapy, or radiotherapy within 4 weeks before the vaccination. The exclusion criteria were (1) active infection, other active malignancy, (2) pregnancy or lactation, and (3) treatment with immunosuppressive agents (e.g., steroids). All patients were informed of the investigational nature of the study and gave written informed consent, in accordance with each institutional review board.

### Treatment protocol

A skin test was performed before the first vaccination by intradermal injection of 10 μg of the peptide, to avoid the risk of acute hypersensitivity. A positive skin reaction was defined as a >30-mm diameter of erythema and induration relative to that of the negative control using saline. The dose was escalated as 0.5, 1, and 3 mg/body of the peptide vaccine. The HIG2-9-4 peptide was emulsified by using incomplete Freund’s adjuvant (Montanide ISA-51 VG, Seppic, Paris, France). The vaccination was given subcutaneously once a week, and four weeks as 1 cycle. After a 1-week interval, the next cycle was performed and continued as a vaccine monotherapy until the judgment of progressive disease (PD) or doctor’s assessment.

### Clinical monitoring and toxicity assessment

Baseline studies included physical examination, blood examination, and CT scan image analysis. All patients were followed up until death, intolerance, or the patient’s withdrawal of consent. Toxicity assessments were performed at least once a week by applying the National Cancer Institute Common Terminology Criteria for Adverse Events version 3.0. Dose-limiting toxicity was defined as a hematological toxicity of grade ≥4, and non-hematologic toxicity of grade ≥3. Clinical and laboratory assessments were checked at each visit.

### Measurement of CTL response in clinical study

IFN-γ enzyme-linked immunospot (ELISPOT) kit and AEC substrate set (BD Pharmingen, San Diego, CA) were used to measure CTL responses in the clinical study, as reported previously [14]. Peripheral blood mononuclear cells (PBMCs) were obtained from patients and were frozen before vaccination and at the end of each course. Frozen PBMCs were thawed and used for in vitro sensitization. Briefly, PBMCs were cultured in 1 mL of complete media (prepared with a mix of AIM-V and RPMI, 50 % of each) containing 10 % fetal bovine serum in a 48-well plate with 10 μg/mL of HIG2-9-4 peptide and 20 IU/mL of IL-2 at 37 °C with 5 % CO_2_, for 2 weeks. On day 7, half of the medium was removed from each well and 500 μL of fresh medium containing epitope peptide, as described above, was added to sensitize. After a 2-week incubation, CD4-positive cells were removed by using a Dynal CD4 positive isolation kit (Invitrogen, Carlsbad, CA, USA), and harvested cells were co-cultured with peptide-pulsed T2 cells (1 × 10^5^ cells per well) at 37 °C for 20 h. HLA-A*0201-restricted HIV-derived epitope peptide (ILKEPVHGV) was used as the control peptide. ELISPOT assay was performed in triplicate. HIG2-9-4-specific CTL response was defined according to an evaluation tree algorithm [15].

## Results

### Patients’ characteristics

A total of nine patients, three each for the 0.5, 1, and 3.0 mg/body cohorts, were enrolled in this trial. The clinical characteristics of these nine patients are shown in Table 1. The performance status was 0 for four patients, 1 for four patients, and 2 for one patient. According to the MSKCC risk criteria, four patients were intermediate risk and five patients were poor risk. Prior to the vaccine treatment, all patients except one had received cytokine therapy (interferon-α or/and interleukin-2), five received TKI treatment (sorafenib and/or sunitinib) in addition to the cytokine therapy and one received sorafenib treatment. The duration of previous treatments varied widely (4–26 months). Six patients had discontinued previous treatment for PD, and three patients had stopped the treatment because of an adverse event (AE). All previous cytokine therapies were discontinued because of PD. The evaluable lesions were lung metastasis in eight patients, pancreatic metastasis in three patients, and local recurrence lesions in two patients.

**Table 1:** Enrolled patient characteristics

Case	Age	Sex	PS	MSKCC criteria	Pre-vaccine treatment	Duration of the previous treatment (months)	Reason of stop the pre-vaccine treatment	Evaluable lesion
1	68	F	0	Intermediate	IFN-α, sorafenib	26	AE	Lung
2	57	M	1	Poor	IFN-α, IL-2, sorafenib	6	PD	Lung
3	50	M	2	Poor	IFN-α, sunitinib	4	PD	Local recurrence
4	74	M	1	Poor	IFN-α, IL-2, sorafenib	10	AE	Lung, pancreas
5	68	M	1	Poor	IFN-α	8	PD	Lung
6	48	M	1	Poor	IFN-α, IL-2, sorafenib, sunitinib	11	PD	Lung, pancreas, local recurrence
7	60	M	0	Intermediate	Sorafenib	10	AE	Lung
8	74	M	0	Intermediate	IFN-α, IL-2	14	PD	Lung, pancreas
9	65	M	0	Intermediate	IFN-α	22	PD	Lung

### Toxicity

No hematologic, cardiovascular, hepatic, or renal toxicities were observed during and after the vaccine therapies (Table 2). The number of vaccinations for each patient ranged from 4 to 88 (average, 37.4). Six patients exhibited RAI, including erythema and induration at the injection sites. RAI was observed when the number of vaccinations was ≥20 times. All of these six patients who exhibited RAI were judged as having stable disease (SD) condition for 4.4–25.9 months (Table 2). On the other hand, two patients with PD at a very high stage showed no sign of RAI. The number of patients in our study was too small to confirm an association between the development of RAI and clinical efficacy.

**Table 2:** Evaluation of adverse event and CTL response

Case	Dosage (mg)	Frequency of vaccination	Hematological	Non-hematological	RAI	CTL response	Timing of the CTL induction	Clinical and imaging evaluation	PFS (months)	OS (months)
1	0.5	88	None	None	Induration	+++	Post 9 co.	SD	25.9	32.1
2	0.5	19	None	None	None	−	–	PD	2.4	5.5
3	0.5	4	None	None	None	+	Post 1 co.	PD	0.9	1.0
4	1.0	45	None	None	Induration	+++	Post 1 co.	SD	8.1	13.1
5	1.0	20	None	None	Erythema	+++	Post 1 co.	SD	4.4	12.1
6	1.0	44	None	None	Erythema	+++	Post 1 co.	SD	10.8	25.8
7	3.0	38	None	None	Induration	+++	Post 1 co.	SD	10.3	77.7
8	3.0	34	None	None	None	+++	Post 2 co.	SD	11.0	68.4
9	3.0	45	None	None	Induration	+++	Post 1 co.	SD	20.8	28.7

### Clinical responses

**Fig. 1:**
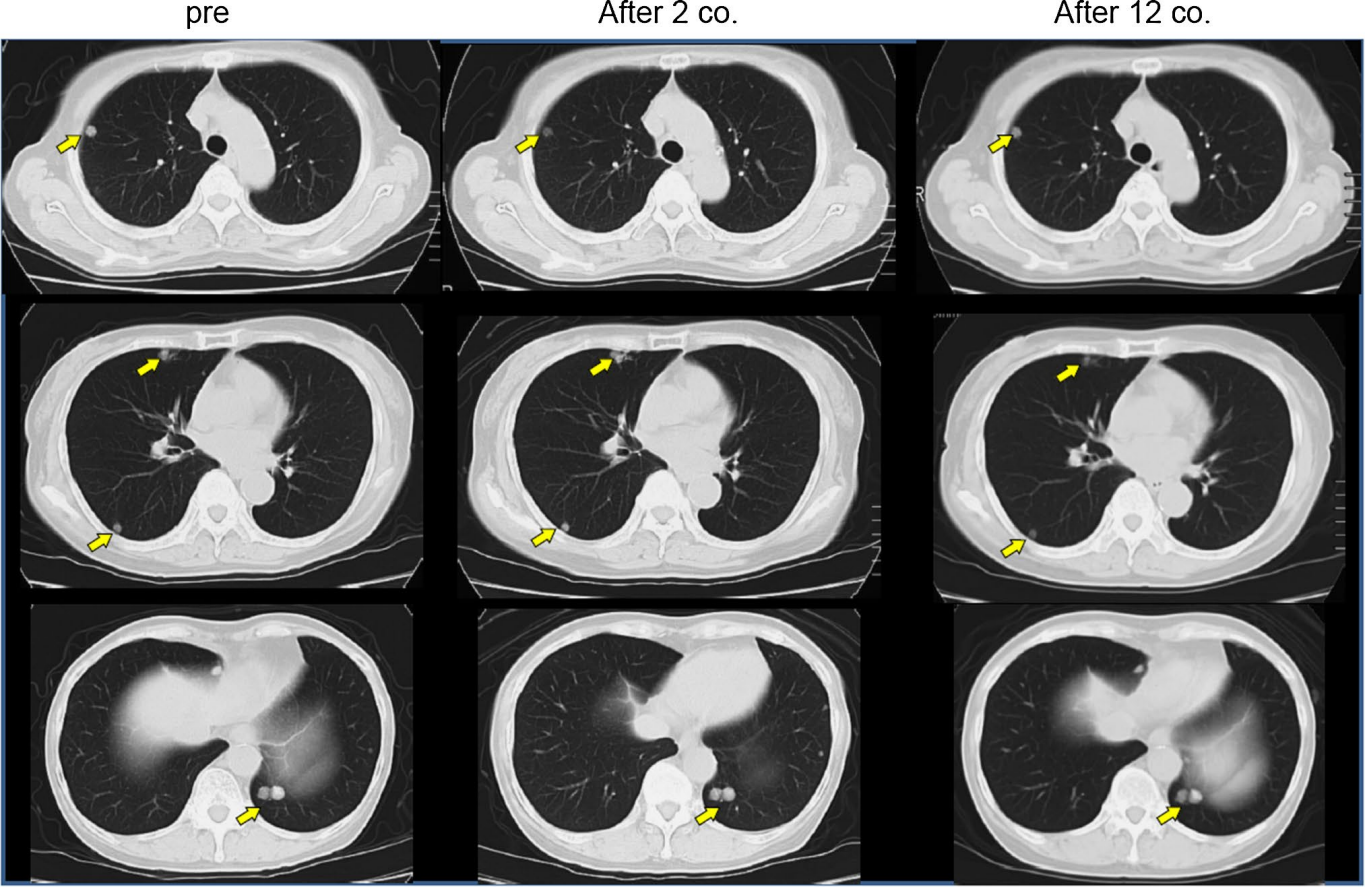
Chest CT image of a long SD case. Chest CT image of case 1, showing multiple lung metastases as indicated by* arrows* before the vaccine treatment. After 2 and 12 courses of peptide vaccine (0.5 mg/body) treatment, the sizes of multiple lung metastases were unchanged

**Fig. 2:**
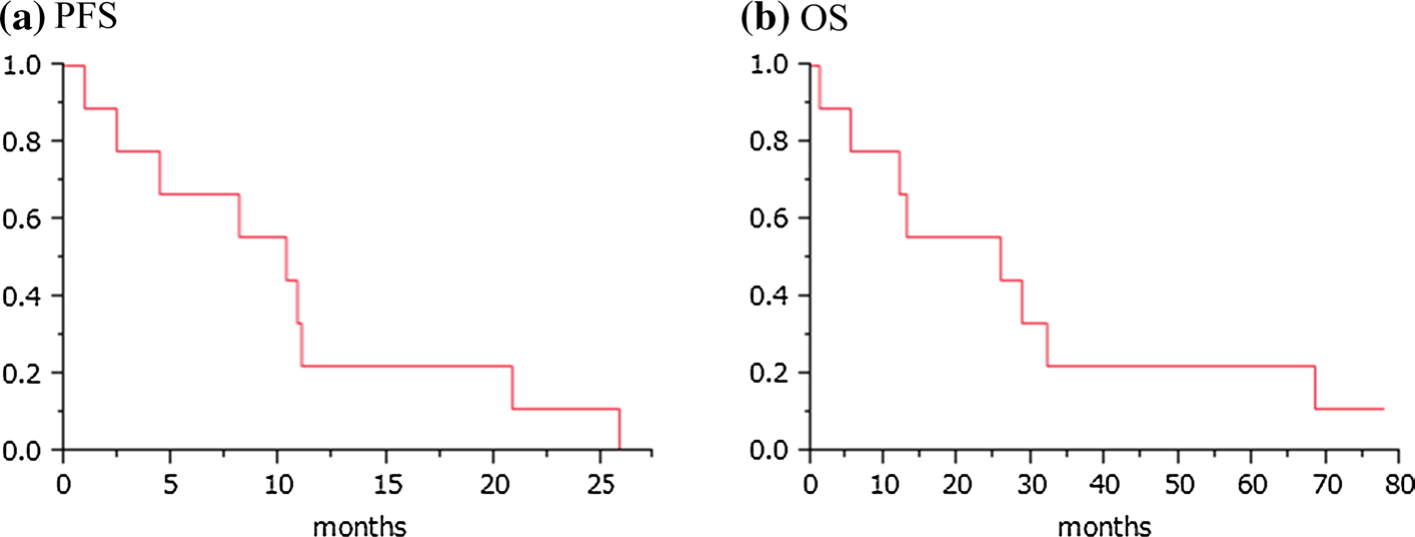
Kaplan–Meier estimates of progression-free survival (**a**) and overall survival (**b**) following HIG2 peptide vaccine therapy. The median PFS and OS are 10.3 and 25.8 months, respectively

Seven patients maintained SD through five courses of vaccination, and the remaining two showed PD within 3 courses, according to the RECIST criteria (Table 2). The disease control rate (all were SD) was 77.8 % in this study. CT scan images of one representative patient with long SD are shown in Fig. 1. Although multiple lung metastases were present at the beginning of the treatment, this patient maintained good performance status and stable condition of these metastatic lesions for nearly 26 months, and showed no signs of severe adverse events, except RAI. The Kaplan–Meier curves for PFS and OS are shown in Fig. 2. The median PFS was 10.3 months, and the median OS was 25.8 months. When patients had undergone a long period of previous treatment, they tended to show longer survival after vaccine therapy.

### CTL response

**Fig. 3:**
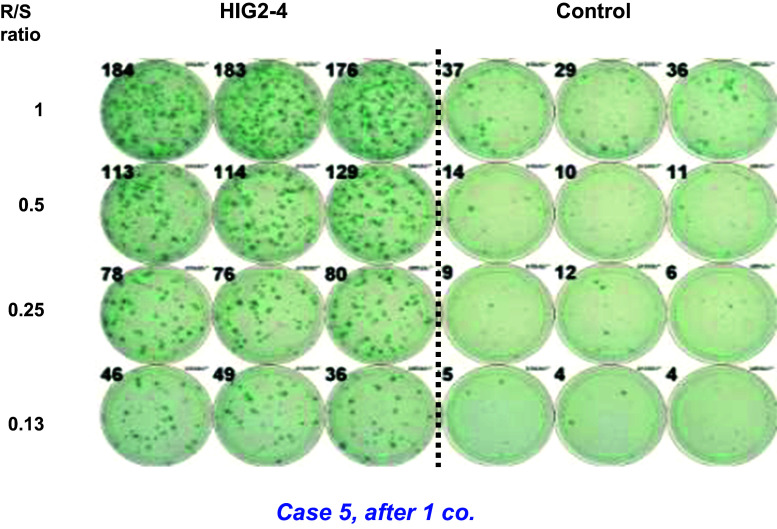
HIG2-9-4-specific CTL response in case 5 after one course of vaccine treatment. The wells in the IFN-γ ELISPOT assay of case 5, after one course of vaccination with HIG2-9-4 peptide. IFN-γ production against HIV-A2 peptide-pulsed T2 cells is demonstrated in control wells. R/S ratio; responder/stimulator ratio

An IFN-γ ELISPOT assay using PBMCs was obtained from the patients to assess CD8^+^ T cell immune responses to the HIG2-9-4 peptide. The representative data of the IFN-γ ELISPOT assay in patient 5 after one course (four injections) of the treatment are shown in Fig. 3. The positive CTL responses were observed in two of the three patients receiving 0.5 mg/body of the vaccination (one showed strong response and the other showed very weak response). All six patients who received 1.0 mg/body or 3.0 mg/body showed strong HIG2-peptide-specific T cell induction (Table 2). Most cases, except for one (patient No. 1), showed CTL induction very early within 1–2 courses after the vaccination. In total, positive CTL responses were observed in eight of the nine patients. Notably, all seven patients who showed strong activation of peptide-specific CTLs maintained SD condition for ≥4 months.

## Discussion

HIG2 has been identified as an oncofetal protein that is expressed highly and specifically in RCC and fetal kidney tissues, which implies that the HIG2 protein could be an attractive molecular target for development of novel treatment for RCC [7]. As one of the approaches to develop a novel treatment option, we previously developed the HLA-A*0201/0206-restricted HIG2-derived epitope peptide, HIG2-9-4 [8]. In the present study, we conducted a phase I clinical trial using the HIG2-9-4 peptide for patients with advanced RCC. The HIG2-peptide vaccine therapy was well tolerated and induced the peptide-specific CTLs very effectively. Among three different dose groups (0.5, 1.0, and 3.0 mg), the peptide-specific CTL responses in the higher dose groups were stronger than those in the 0.5-mg peptide group, although the number of patients was too small to draw a definite conclusion. However, since no dose-limiting toxicities were observed, we suggest that the optimal dose of this peptide for further clinical trials is 3.0 mg/body.

Among the nine patients enrolled in this study, strong peptide-specific CTL responses were induced in seven patients, all of whom maintained SD for ≥4 months. However, two patients with no or weak immune response developed PD very quickly. This result might suggest that induction of HIG2-9-4 peptide-specific CTLs could contribute to better clinical outcomes.

When our clinical trial started, the molecular-targeted therapies using sorafenib and sunitinib for mRCC had just been approved in our country. Therefore, the patients who enrolled in this trial received cytokine therapy and/or TKI therapy before enrollment. A phase III study of sorafenib for patients with cytokine-refractory advanced RCC reported a median PFS of 5.5 months [18]. Moreover, a phase III study of sunitinib for RCC patients who had no previous systemic therapy reported a median PFS of 11.0 months [19]. The median PFS in our study was calculated to be 10.3 months. Our result was consistent with the results of sorafenib and sunitinib, although tumor shrinkage was not observed in any of the nine patients treated with the HIG2 vaccine. In this study, none of the enrolled patients had liver, bone, or brain metastases. We might consider this group of RCC patients as ideal for peptide vaccine therapy.

Several RCC-associated antigens as well as HLA-class I-restricted epitope peptides have been previously reported [20]. However, only a limited number of clinical studies using the peptide-based vaccine for RCC have been reported [21–27], and the clinical benefit of vaccine therapy for RCC is likely to be limited to a small subset of patients. Walter et al. reported phase I/II clinical trials using multiple tumor-associated peptides (TUMAPs called IMA901) [28]. They treated a total of 96 HLA-A02-positive patients with mRCC, using IMA901. In the phase I part, they showed an association of T-cell responses to multiple TUMAPs with better disease control. In the randomized phase II part, they demonstrated that cyclophosphamide administration before IMA901 reduced the number of regulatory T cells (Tregs), and that immunological response of the patients to multiple TUMAPs was associated with longer survival. However, a randomized phase III study to confirm the clinical benefit was reported to have given negative results more recently. A G250 monoclonal antibody has also been identified for an RCC-associated antigen, which was later proven to be identical to carbonic anhydrase protein (CA IX) [29]. A Phase III clinical trial as an adjuvant treatment of clear-cell RCC after nephrectomy demonstrated a significant benefit of prolonged disease-free survival in patients with high CA IX score in the resected RCC, compared with patients in the placebo [30]. Cancer immunotherapy, including peptide vaccine therapy, may be more effective for patients with a postoperative adjuvant therapy or minimum disease status.

Molecular-targeted therapy is effective in advanced RCC, but a large proportion of patients experience various severe AEs. These include hypertension, hand–foot skin reaction, fatigue, diarrhea, anemia, and thrombocytopenia, which decrease the quality of life for patients and may sometimes lead to life-threatening conditions. On the other hand, although the peptide vaccine treatment does not cause any severe systemic AEs in general, only a subset of patients can expect a clinical benefit. In addition, at present, immune checkpoint blockades, such as anti-PD-1/PD-L1 or anti-CTLA-4 antibodies, are considered to be the most promising drugs for treatment of advanced cancer patients. For this type of treatment, the presence of CTLs that recognize a cancer-specific antigen(s) with an HLA class I molecule on cancer cells is now suggested to be a critical predictor of better clinical outcomes. Particularly, CTLs recognizing neoantigens that are generated by the somatic mutations that occur in cancer cells are considered to be strong inducers for CTLs in cancer tissues [31, 32].

However, CTLs specifically recognizing oncoantigen peptides such as HIG2, which are broadly expressed in cancer cells but not in normal cells, may also contribute to the clinical outcome of immune checkpoint blockade therapies. At this moment, although neoantigens are certainly more cancer-specific than oncoantigen-derived peptides, we still do not know which induce a higher level of anti-tumor immune response in cancer patients. In addition, it is also certain that HLA-restricted cancer peptide vaccines derived from oncoantigens can be more widely applied to a larger subset of cancer patients, than can individualized neoantigens. We suspect that the combination of an immune checkpoint blockade with CTL-inducing active immunotherapy and either neoantigens or oncoantigens could enhance the clinical benefit.

In summary, HIG2-9-4 peptide vaccine treatment was tolerable and effectively induced peptide-specific CTLs in RCC patients. This novel peptide vaccine therapy for RCC appears to be promising.

## Abbreviations

AE: Adverse event
BIMAS: BioInformatics and molecular analysis section
BLAST: Basic local alignment search tool
CT: Computed tomography
CTL: Cytotoxic T lymphocyte
ECOG: Eastern Cooperative Oncology Group
ELISPOT: Enzyme-linked immunospot
GMP: Good manufacturing practice
HIG2: Hypoxia-inducible protein 2
HLA: Human leukocyte antigen
IFN: Interferon
OS: Overall survival
PBMC: Peripheral blood mononuclear cell
PD: Progressive disease
PFS: Progression-free survival
RAI: Reactions at the injection sites
RCC: Renal cell carcinoma
RECIST: Response evaluation criteria in solid tumors
SD: Stable disease
TKI: Tyrosine kinase inhibitor
Treg: Regulatory T cell
TUMAP: Tumor-associated peptide
